# Development of Case Numbers during the COVID-19 Pandemic in a Center of Maximum-Care for Traumatology and Orthopedic Oncology

**DOI:** 10.3390/healthcare9010003

**Published:** 2020-12-22

**Authors:** Christina Polan, Manuel Burggraf, Max Daniel Kauther, Heinz-Lothar Meyer, Friederike Rademacher, Henrik Braitsch, Karl-Heinz Jöckel, Jendrik Hardes, Arne Streitbürger, Marcel Dudda

**Affiliations:** 1 Department of Trauma, Hand and Reconstructive Surgery, University Hospital Essen, Hufelandstraße 55, 45147 Essen, Germany; Manuel.Burggraf@uk-essen.de (M.B.); MaxDaniel.Kauther@uk-essen.de (M.D.K.); Heinz-Lothar.Meyer@uk-essen.de (H.-L.M.); Friederike.Rademacher@uk-essen.de (F.R.); Marcel.Dudda@uk-essen.de (M.D.); 2 Central Department of Medical Controlling, University Hospital Essen, Hufelandstraße 55, 45147 Essen, Germany; Henrik.Braitsch@uk-essen.de; 3 Institute of Medical Informatics, Biometry and Epidemiology, University Hospital Essen, Hufelandstraße 55, 45147 Essen, Germany; k-h.joeckel@uk-essen.de; 4 Department of Tumor Orthopedics, University Hospital Essen, Hufelandstraße 55, 45147 Essen, Germany; Jendrik.Hardes@uk-essen.de (J.H.); Arne.Streitbuerger@uk-essen.de (A.S.)

**Keywords:** COVID-19, coronavirus, pandemic, emergency medicine, orthopedics, public health, case numbers, hospital capacity

## Abstract

(1) Background: The COVID-19 pandemic has led to a significant change in the utilization of trauma surgery and tumor orthopedic hospital facilities. (2) Methods: In a monocentric retrospective analysis, the weekly numbers of cases requiring intra-clinical treatment in the first four months of 2020 were compared with those of 2019. Patients’ visits to the emergency department and shock room, consultation hours, work-related accidents, case numbers in the normal and intensive care units, ventilation hours, the “Simplified Acute Physiology Score/ Therapeutic Intervention Scoring System” (SAPS/TISS), the average length of stay in hospital, the number of operations and their degree of urgency, as well as deaths, were analyzed in a study based on the data from 7606 outpatient consultations in 2019 and 6755 in 2020, as well as 993 inpatient cases in 2019 and 950 in 2020. (3) Results: There was a significant reduction in the number of treatments per week in the emergency department (261 ± 29 vs. 165 ± 25; *p* < 0.001) with the same number of shock room treatments and fewer consultation hour contacts (226 ± 29 vs. 119 ± 65; *p* = 0.012). There were fewer inpatient cases (66 ± 7 vs. 42 ± 11; *p* = 0.001), resulting in a fall in the days of hospitalization (492 ± 63 vs. 308 ± 78; *p* < 0.001) and number of operations (73 ± 7 vs. 55 ± 10; *p* = 0.012), especially elective procedures (20 ± 3 vs. 7 ± 7; *p* = 0.008). The SAPS/TISS score was lower (1351 ± 1213 vs. 399 ± 281; *p* = 0.023). Fewer fracture treatments and septic surgeries were performed, while the number of procedures to treat orthopedic malignancies remained constant. (4) Conclusions: During the first phase of the COVID-19 pandemic, we observed a significant reduction in the number of cases treated in orthopedics. While the number of multiple-injured patients was unchanged, fewer patients presented for primary and regular care. Treatment of acute injuries and malignant tumor diseases was not at risk. There was no effect on in-house mortality. We see a potential for the recruitment of medical staff from the outpatient department, operating room, and the ward. In the event of a future second wave, our results may allow for early planning, particularly of the all-important human resources. Reorganization by hospitals and decreased patient numbers in trauma surgery can enable the reallocation of medical staff, equipment, and beds to increase capacity for COVID-19 patients.

## 1. Introduction

In late December 2019 there was an outbreak of a viral disease, now known as COVID-19, in Wuhan, China, which first spread throughout the country and was then declared a worldwide pandemic by the World Health Organization (WHO) [1,2,3]. On 18 November 2020, 55,714,647 people worldwide were registered as infected and 1,340,645 as deceased [4]. In Germany, the virus began to spread on 27 January 2020, and by 18 November, 843,757 cases of infection had been registered. Drastic measures with a first lockdown were introduced in the thirteenth calendar week in March 2020 to inhibit the pandemic. Both the virus and the measures taken to combat it have had a serious direct impact on society, national economies, and everyday life [5]. The pandemic also created special challenges for German hospitals, not only in the treatment of infected patients but also in other departments where the effect of the pandemic on patient numbers became clearly noticeable [6]. For example, fewer heart attacks were reported in cardiology, and fewer strokes in neurology [7,8,9].

On the other hand, psychiatric illnesses such as sleep disorders, depression, and anxiety disorders occurred more frequently during the lockdown, a phenomenon that may also influence the suicide rate [10,11]. Furthermore, with families having to stay in close contact at home all day, there has been an increase in domestic violence and abuse of children and partners [12,13,14]. In many centers, the number of therapies for musculoskeletal tumors, especially benign tumors, decreased during the corona pandemic [15]. In an online survey, orthopedic, and trauma surgery professors in Germany reported a significant reduction in surgical capacity during the COVID-19 pandemic, with a corresponding reduction in revenue [16].

The official restrictions in the German health care system started in week 11 with an appeal to recruit staff and postpone scheduled surgeries and procedures. In week 12, an official measure and coordination office for unoccupied intensive care beds were established, and from week 15 onwards, it became mandatory to report all available Intensive Care Unit (ICU) beds [5].

At our university hospital, there was a reduction in the surgical capacity of up to 50% from the twelfth week, which began to return to normal from 8 June 2020.

There is now some debate about how the patient population changed during the pandemic, whether the provision of surgical care was at risk and whether this had an impact on hospital lethality. This study examines the development in weekly case numbers in the sectors of trauma surgery and orthopedic tumor care in a maximum-care hospital during the COVID-19 pandemic.

## 2. Materials and Methods 

### 2.1. Study Design and Data Collection

In this monocentric, retrospective data analysis, the development of case numbers in a maximum-care center for trauma surgery was recorded for the shock room, emergency department, consultation hours, operation theatre, normal and intensive care units. The second to seventeenth weeks in the first third of 2020 were compared with the same period of the previous year. Two phases were defined, corresponding to the second to tenth weeks and the eleventh to seventeenth weeks, while intra-clinical regulations and measures were taken in response to the COVID-19 pandemic. 

### 2.2. Ethics

The study was approved by the Ethics Committee of the Medical Faculty of the University of Duisburg-Essen with the sign 20-9285-BO. 

### 2.3. Examination Parameters

The outpatient case numbers of the emergency department, consulting hours, and work-related accidents were recorded. The total number of surgical interventions, including elective interventions and interventions in different sub-areas, were determined, and on-call operations, were evaluated. In addition, the number of trauma shock room treatments, the average hospital stay, the occupancy days in the normal and intensive care units, the SAPS/TISS scores for patients in intensive care units, ventilation hours, and hospital lethality were included in the analysis.

### 2.4. Statistical Analysis

The SPSS 25 software was used for statistical analysis (IBM, New York, NY, USA). Within the context of descriptive statistics, a calculation of the mean value, the standard deviation, the median, the interquartile range, and the two-sided 95% confidence interval was performed. In addition, the percentual increase or decrease in the mean values over the two phases was calculated and compared. All values were tested for normal distribution. For non-normally-distributed values, the non-parametric Mann–Whitney-U test was used to detect differences between unconnected test groups *p* < 0.05 was defined as statistically significant.

## 3. Results

To the best of our knowledge, there was no evidence of COVID-19 infections among any of the patients, nursing staff, or doctors in our department in the first phase of the COVID-19 pandemic. Seventeen nurses and five doctors observed a two-week quarantine as asymptomatic contact persons without proof of illness. Four nurses were assigned to other units or to help set up a COVID-19 emergency department. Three physicians were deployed to support preclinical medicine, especially COVID-19 diagnostics. Altogether, data from 7606 outpatient consultations in 2019 and 6755 in 2020, as well as 993 inpatient cases in 2019 and 950 in 2020, were analyzed.

### 3.1. Emergency Department and Shock Room

**Figure 1:**
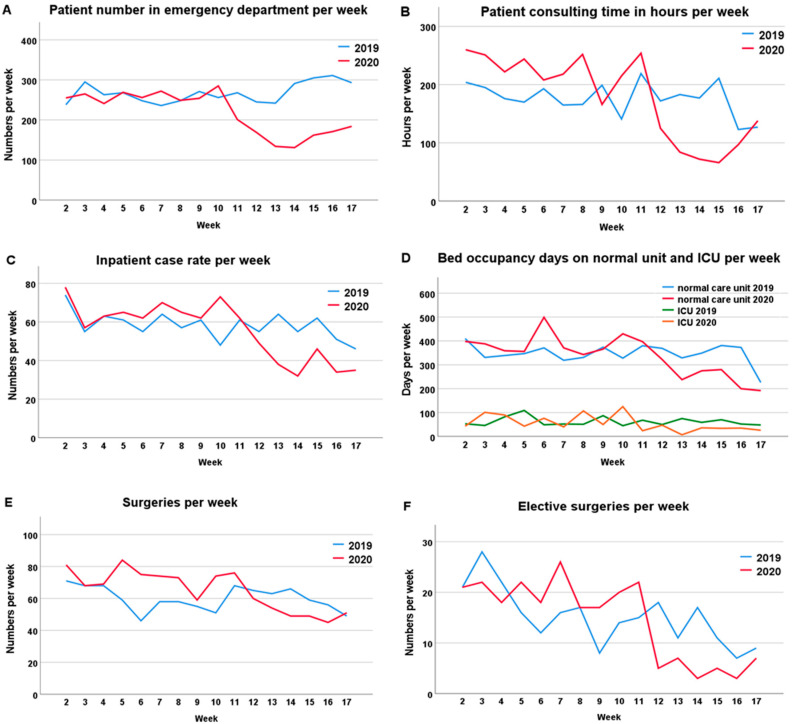
Time course of patient presentations, surgeries, and inpatient case numbers. The data of the 2nd to 17th weeks in 2019 (blue) and in 2020 (red) were compared. From the 11th week onwards, a decrease in case numbers could be detected, which could be attributed to the COVID-19 pandemic. (**A**) Patient numbers in the emergency department per week; (**B**) Patients consulting hours per week; (**C**) Stationary case rates per week; (**D**) Bed occupancy days on the normal and intensive care units per week; (**E**) Number of surgeries per week; (**F**) Number of elective surgeries per week.

**Figure 2:**
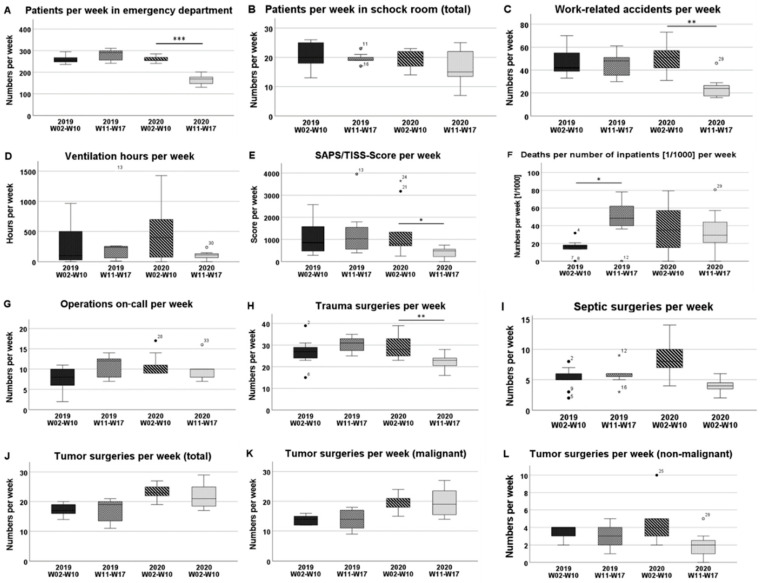
Comparison of the time phases in 2019 and 2020. The boxplots represent the investigated parameters in weeks 02–10 and in weeks 11–17 in 2019 and in 2020. The significance levels are defined as * *p* < 0.05, ** *p* < 0.01, and *** *p* < 0.001. (**A**) Patient numbers per week in the emergency department; (**B**) Patient numbers per week in the shock room; (**C**) Work-related accidents per week; (**D**) Ventilation hours per week; (**E**) SAPS/TISS-Score per week; (**F**) Deaths per number of inpatients (1/1000) per week; (**G**) Operations on-call per week; (**H**) Trauma surgeries per week; (**I**) Septic surgeries per week; (**J**) Musculoskeletal tumor surgeries in total per week; (**K**) Malignant or uncertain musculoskeletal tumor surgeries per week; (**L**) Non-malignant musculoskeletal tumor surgeries per week.

From the eleventh week of 2020 onwards, there was a decrease of 37% (261 ± 13 vs. 165 ± 25; *p* < 0.001) in the number of admissions per week in the emergency department (Table 1), (Figure 1A and Figure 2A).

In the previous year, no significant difference was detected for the same period. There was no statistically significant difference in the number of shock room treatments between the two phases examined (19 ± 3 vs. 17 ± 6; *p* = 0.470), and there was also no difference in comparison with the previous year. However, between the twelfth and fourteenth weeks of 2020, after the lockdown period had begun, there was a significant decline in the number of shock room treatments (Figure 2B). The total number of work-related accidents presenting in the emergency room in weeks 11 to 17 decreased by 51% compared to the numbers in weeks 2 to 10 (51 ± 12 vs. 25 ± 10; *p* = 0.001) (Figure 2C).

### 3.2. Consulting Hours

In the outpatient department of traumatology, there was also a 47% decrease in the number of patients presenting for examination in the second phase of the first third of 2020 (226 ± 29 vs. 119 ± 65; *p* = 0.012) (Figure 1B). 

### 3.3. Normal and Intensive Care Units

While the average length of hospital stay was not affected, the number of inpatient cases decreased by 36% during the COVID-19 pandemic (66 ± 7 vs. 42 ± 11; *p* = 0.001) (Figure 1C). The total number of weekly occupancy days fell by 30% in the normal care unit, while it decreased by 60% in the intensive care unit (Figure 1D). In the intensive care setting, the SAPS/TISS scores were lower by 70% (1351 ± 1213 vs. 399 ± 281; *p* = 0.023) and a 77% reduction in ventilation hours was observed (455 ± 480 vs. 106 ± 78; *p* = 0.210) (Figure 2D,E). Overall, there was no significant difference in the “in-house mortality rate” of our department during the pandemic period (Figure 2F).

### 3.4. Operations

Comparison of the two phases showed that from the eleventh week onwards, the total number of operations fell by 25% from 73 ± 7 per week before the pandemic to 55 ± 10 (*p* = 0.012) (Figure 1E). Elective operations were the most heavily affected. These fell by 65% from 20 ± 3 to 7 ± 7 per week (*p* = 0.008) (Figure 1F). In the previous year, there was no significant difference in the same time periods. The number of on-call surgeries did not have to be increased despite reduced capacity in the operating theatres (Figure 2G). In tumor orthopedics, 8% fewer operations were performed from the eleventh week onwards. While operations on benign tumors decreased by 52% (4.2 ± 2.4 vs. 2 ± 1.7; *p* = 0.055), the number of operations on malignant tumors or neoplasms of uncertain behavior remained approximately the same and even rose by 5% (19 ± 3 vs. 20 ± 5; *p* = 1.000). Compared to the previous year, the number of operations per week increased (Figure 2J–L). The number of trauma treatments per week decreased by 27% (30 ± 6 vs. 22 ± 4; *p* = 0.008) (Figure 2H) and interventions in septic surgery decreased by 56% (9 ± 3 vs. 4 ± 1; *p* = 0.002) (Figure 2I).

## 4. Discussion

The corona pandemic has been a challenge not only to departments providing primary care for internal medicine, but it has also had an enormous impact on the development of case numbers in less directly affected departments such as trauma surgery and orthopedics.

Interestingly, there was a significant decrease of 37% in patient contacts in the emergency department. This may be explained by the reduced volume of road traffic, social distancing, abstention from team sports or high-risk sports, or avoidance behavior due to fear of infection with COVID-19. This directly reflects the effect of the administrative order of a lockdown. Similar explanations have been given in current studies on the effect of the lockdown on hand and upper limb emergencies [16], emergency department radiology volumes [17], and patterns of adult and pediatric hand trauma [18].

In the context of the pandemic, people may also have attached less importance to minor injuries such as bruises or cuts or chronic conditions, which were probably treated by unsupervised self-healing or in an outpatient setting. Since no adverse effects due to delayed or non-treatment have so far been discovered in our patient population, it can be concluded from our experience during the pandemic period that the threshold for using our trauma surgery emergency room could be raised without endangering patients. We will further investigate the outcome of delayed patient presentation, for example, regarding incorrectly healed fractures or chronic inflammation. It would be advisable, especially as the pandemic continues, to inform and encourage patients to go to hospital with serious injuries and signs of infection.

We found no significant difference in the number of cases in shock room treatments. Nevertheless, a pronounced low point in weeks 12 to 14 was possibly due to reduced risk behavior and the lower volume of road traffic in the context of the lockdown. This is in line with the early stage of the pandemic in China, during which a reduction in the number of injuries due to traffic accidents and outdoor activities was registered [19].

The decline in consultation hour contacts of 47% can be explained by the cancellation of all elective appointments, but also by a reduction in the number of appointments requested and early care by office-based colleagues and by patients not attending due to avoidance behavior. Reduced case numbers, as well as reduced occupancy days, might be due to the cancellation of many complex elective procedures, but also to the lower number of patients in the emergency department. Here, too, a critical investigation is necessary to determine whether follow-up treatment and follow-up examinations could be shifted into the outpatient sector. In the intensive care setting, there was a marked reduction in the SAPS/TISS scores, a reduction in ventilation hours due to the reservation of monitored beds for COVID-19 patients, and lower bed utilization. Here, capacities might be available for COVID-19 patients requiring intensive care or ventilation. An increased weekly in-house mortality of trauma surgery and orthopedic tumor patients was not present in our study.

The total number of operations decreased by 25%, and elective procedures dropped by 65% due to cancellation by the hospital. Nevertheless, the treatment of fractures, infections, and malignant tumors was not jeopardized at any time and was carried out mainly during normal working hours outside shift times.

In summary, there was an extraordinary decrease in the number of patients in orthopedics during the first phase of the COVID-19 pandemic. There was no overburdening of medical staff during normal duty hours because there were fewer patients, and the number of on-call operations remained the same. Therefore, patients’ access to surgical treatment of emergency trauma and malignant orthopedic tumors was fully guaranteed at all times despite bed closures and the reduction in operating capacity at our level-1 trauma center during the COVID-19 pandemic. Our data confirmed the assessments of a survey of 52 German clinical directors regarding the expected substantial financial and personnel changes in the orthopedic and trauma surgery departments of university hospitals impacted by the COVID-19 pandemic. They expected an average reduction of 49.4% in operating room capacity, 29.3% loss of revenue, and a reallocation of 14.7% of physicians from orthopedic and trauma surgery to other care-delivery environments [20].

Some changes in the case numbers can also be explained by measures taken by hospitals or the state. All elective procedures were canceled. Similarly, elective consultation appointments were canceled, and outpatient wound controls were shifted to the ambulatory medical sector, resulting in fewer patients presenting to the emergency department. These recommendations were also issued for Italy [21] and presented in a series of measures regarding hospital perioperative preparation recommended by the European Society of Trauma and Emergency Surgery (ESTES) [22]. The cancellation of major elective surgeries also led to a decrease in trauma surgery ICU bed utilization, allowing deployment of ICU staff to other units. The decline in inpatient case numbers by 36% can be explained by the early discharge of patients to home care and the non-admission of elective patients in order to save bed capacity.

In addition, the treatment of non-infected patients and COVID-19-positive patients were segregated with sections of emergency, normal, and intensive care units reserved exclusively for COVID-19 patients. Avoidance of personal contact and social distancing regulations were introduced, and the wearing of personal protective equipment was mandatory throughout the hospital area. Business travel was canceled for all employees. Scientific staff were allowed to work via home offices, and group meetings were replaced by online meetings. Patients were not allowed to receive visitors in order to minimize the number of persons in the building and thus protect staff and patients from the risk of infection.

With rising infection rates in the second phase of the COVID-19 pandemic in Europe and a change in risk behavior of the population, future studies will investigate how orthopedic patient numbers have developed in the further course.

## 5. Conclusions

During the first phase of the COVID-19 pandemic, a significant decrease in the number of orthopedic outpatient and inpatient cases was observed. Fewer patients presented for primary and regular care and treatment of work-related accidents, which we believe is directly linked to the lockdown. On the other hand, the number of multiple-injured patients and orthopedic tumor patients remained unchanged. It can be concluded that even in case of massive administrative interference, adequate resources must be kept available for these patient groups. The treatment of seriously injured patients and tumor patients with malignant diseases was guaranteed at all times. We see a potential for the recruitment of medical staff from the outpatient department, operating room, and the wards. In the event of a future second wave, our results may allow for early planning, particularly of the all-important human resources.

Reorganization by hospitals and decreased patient numbers in trauma surgery can enable reallocation of medical staff, equipment, and beds to increase capacity for COVID-19 patients.

## Figures and Tables

**Table 1:** Descriptive statistics of the investigated consultations and cases with mean ± standard deviation, 95% confidence interval, and *p*-values of weeks 02–10 vs. weeks 11–17 of 2019 and 2020. Statistical difference of the non-paired and non-parametric values was determined by the Mann–Whitney U test. In addition, the difference in the mean values between weeks 02–10 vs. weeks 11–17 was determined as ∆ (%) of W02–W10.

Numbers per Week	2019				2020			
	W02–W10	W11–W17	*p*-Value	∆ (%)	W02–W10	W11–W17	*p*-Value	∆ (%)
Patients emergency department	258 ± 19	279 ± 28	0.174	8	261 ± 13	165 ± 25	<0.001	−37
	(244; 272)	(253; 305)			(250; 271)	(141; 188)		
Shock room treatment	20 ± 5	20 ± 2	0.758	0	19 ± 3	17 ± 6	0.470	−11
	(17; 24)	(18; 21)			(17; 22)	(11; 23)		
Work-related accidents	46 ± 12	45 ± 12	0.918	−2	51 ± 12	25 ± 10	0.001	−51
	(37; 55)	(34; 55)			(42; 61)	(15; 34)		
Patients in consultation hours	179 ± 21	173 ± 37	1.000	−3	226 ± 29	119 ± 65	0.012	−47
	(163; 195)	(139; 208)			(204; 249)	(60; 180)		
Inpatient case numbers	60 ± 7	56 ± 7	0.408	−7	66 ± 7	42 ± 11	0.001	−36
	(54; 65)	(50; 62)			(61; 71)	(32; 52)		
Mean hospital stay (days)	7.2 ± 0.9	6.8 ± 0.6	0.252	−6	7.7 ± 1.6	6.7 ± 1.9	0.114	−13
	(7; 8)	(6; 7)			(6; 9)	(5; 9)		
Inpatient occupancy (days) (total)	420 ± 39	421 ± 63	0.606	0	492 ± 63	308 ± 78	<0.001	−37
	(390; 450)	(362; 479)			(444; 541)	(236; 380)		
Inpatient occupancy (days) (normal care)	350 ± 29	344 ± 55	0.606	−2	390 ± 48	272 ± 72	0.005	−30
	(327; 373)	(293; 395)			(353; 427)	(206; 338)		
Inpatient occupancy (days) (ICU)	64 ± 23	60 ± 11	0.837	−6	75 ± 32	30 ± 13	0.001	−60
	(46; 81)	(50; 70)			(50; 100)	(18; 41)		
SAS/TISS-Score	1148 ± 820	1371 ± 1242	0.758	19	1351 ± 1213	399 ± 281	0.023	−70
	(517; 1778)	(223; 2520)			(419; 2283)	(139; 659)		
Ventilation hours	292 ± 349	340 ± 523	0.981	16	455 ± 480	106 ± 78	0.210	−77
	(24; 560)	(−144; 823)			(86; 824)	(34; 178)		
Deaths per inpatients (1/1000)	15 ± 10	47 ± 25	0.012	213	36 ± 27	34 ± 27	1.000	−9
	(7; 23)	(24; 70)			(15; 56)	(10; 59)		
Total number of surgeries	59 ± 8	61 ± 7	0.758	3	73 ± 7	55 ± 10	0.012	−25
	(53; 66)	(55; 67)			(67; 79)	(45; 65)		
Elective surgeries	17 ± 6	13 ± 4	0.142	−24	20 ± 3	7 ± 7	0.008	−65
	(13; 22)	(9; 16)			(18; 22)	(1; 14)		
Operations on-call	8 ± 3	11 ± 3	0.091	38	11 ± 3	10 ± 3	0.536	−9
	(5; 10)	(8; 13)			(9; 13)	(7; 13)		
Tumor surgeries (total)	17 ± 2	17 ± 4	0.918	0	24 ± 3	22 ± 5	0.408	−8
	(16; 19)	(13; 21)			(22; 26)	(18; 26)		
Tumor surgeries (non-malignant)	3.4 ± 0.9	3 ± 1.5	0.536	−12	4.2 ± 2.4	2 ± 1.7	0.055	−52
	(3; 4)	(2; 4)			(2; 6)	(0; 4)		
Tumor surgeries (malignant)	14 ± 2	14 ± 4	0.837	0	19 ± 3	20 ± 5	1.000	5
	(13; 15)	(10; 17)			(17; 22)	(15; 24)		
Trauma surgeries	27 ± 6	30 ± 4	0.174	11	30 ± 6	22 ± 4	0.008	−27
	(22; 32)	(27; 34)			(25; 34)	(19; 26)		
Septic surgeries	5 ± 2	6 ± 2	0.536	−20	9 ± 3	4 ± 1	0.002	−56
	(4; 7)	(4; 8)			(6; 11)	(3; 5)		
